# Obituary Notices

**Published:** 1848-06

**Authors:** 


OBITUARY NOTICES.

*Died*, in Princeton, Ky., on Saturday, April 29, 1848, Dr. James E. Throckmorton.

Dr. Throckmorton was one of the oldest and most influential members of the profession in south-western Kentucky. He was an active and successful practitioner for about forty years, and during that long period sustained the character of a learned physician and a liberal and accomplished gentleman.

*Died,* in this city, on Saturday, May 5, 1848, after a short illness, Dr. W. N. Miller, aged 27 years.

Dr. Miller was a capable and skilful physician, and was rapidly acquiring the confidence of the community when he was thus cut off. He was upright and courteous in his bearing towards his brethren, zealously devoted to the profession, and ready and prompt in the discharge of his professional and social duties. He left a wide circle of friends to deplore his early death.

*Died,* suddenly, in this city, on Saturday, May 27, 1848, Doctor E. Lindenheim.

Dr. Lindenheim was a native of Germany, and received his education at the Frederic-William University of Berlin. He had resided in this city about two years, and had made many friends both among the foreign and native population.

Dr. L. H. Mosby, long a citizen of Louisville, and an old and highly respectable practitioner of physic, died at the residence of his daughter, Mrs. McCutchen, in Mississippi, on the 6th of May, in the 58th year of his age. It has not fallen to the lot of the writer of this notice to know a more conscientious or amiable individual than the deceased, and he deems it a simple act of justice to his memory thus to bear testimony to his virtues. Dr. Mosby was a Virginian by birth and education, and possessed all the warmth of feeling supposed to belong to southern character; but his ardor was in the social and gentler feelings, for he seemed to us as free as men ever become in this world from all that is termed passion. He possessed an active and inquisitive mind, which was improved by a thorough preliminary and professional education, and but for an unfortunate impediment in his speech, and infirm health, he would assuredly have attained to eminence in his profession. He was post-master at Louisville during the administration of Mr. Tyler, and for many years was too feeble to perform the labor of a large practice. He died, as he had lived, at peace with all men, and in full assurance of the hopes of the gospel.

